# Sex-Specific Effect of Obesity on Epiblepharon in Different Age Groups: A Case-Control Study

**DOI:** 10.3390/ijerph191912839

**Published:** 2022-10-07

**Authors:** Jin-Jhe Wang, Chien-Hsiung Lai, Ting-Yu Kuo, Meng-Hung Lin, Yao-Hsu Yang, Chau-Yin Chen

**Affiliations:** 1 Department of Ophthalmology, Chiayi Chang Gung Memorial Hospital, Chiayi 61363, Taiwan; 2 Department of Ophthalmology, College of Medicine, Chang Gung University, Taoyuan 33302, Taiwan; 3 Department of Nursing, Chang Gung University of Science and Technology, Chiayi 61363, Taiwan; 4 School of Traditional Chinese Medicine, College of Medicine, Chang Gung University, Taoyuan 33302, Taiwan; 5 Health Information and Epidemiology Laboratory, Chang Gung Memorial Hospital, Chiayi 61363, Taiwan; 6 Department of Traditional Chinese Medicine, Chang Gung Memorial Hospital, Chiayi 61363, Taiwan

**Keywords:** epiblepharon, body mass index, obesity, overweight, sex-specific difference

## Abstract

Obesity has been regarded as a risk factor for several ocular diseases. This study aims to investigate the age- and sex-specific relationship between epiblepharon and obesity in children. A retrospective case–control study was conducted using the Chang Gung Research Database. Children ≤ 18 years of age with epiblepharon were identified from 1 January 2009 to 31 December 2019. Children were classified into three groups: normal, overweight and obese groups. A total of 513 patients and 1026 controls (57.7% males) aged 1 to 18 matched by sex and age were included in the analysis. The median body mass index (BMI) of children with epiblepharon was significantly higher than that of children without epiblepharon (*p* < 0.001). In the subgroup analysis, among boys aged 4 to 9 years, the BMI in boys with epiblepharon was significantly higher than that in boys without epiblepharon (*p* < 0.05) and the risk of epiblepahron in overweight/obese boys was significantly higher than in non-overweight boys (OR = 1.74, 95% CI = 1.07–2.82 for age 4 to 6; OR = 3.06, 95% CI = 1.56–6.03 for age 7 to 9). On the other hand, among girls aged 13 to 18 years, the BMI in adolescent girls with epiblepharon was significantly higher than that in the control group (*p* < 0.05) and overweight/obese girls had a statistically higher risk of persistent epiblepharon than non-overweight girls (OR = 3.70, 95% CI = 1.38–9.97). The association between obesity and epiblepharon varies in strength according to age in a sex-specific manner.

## 1. Introduction

Epiblepharon is a common congenital structural abnormality of the eyelid characterized by excess horizontal folds of skin extending over the margin of the eyelid that misdirect the eyelashes towards the ocular surface [1]. This eyelid anomaly is typically encountered in East Asian descendants with an incidence of approximately 9–10% [2,3,4]. Children with epiblepharon are often subject to irritation, tearing and photophobia, hence presenting with frequent blinking, eye rubbing, and in more severe cases, corneal erosion [2]. Severe keratopathy can induce refractive errors, which may ultimately lead to permanent visual impairment, particularly during the critical period of visual development [5,6]. The main etiology of epiblepharon is considered the distinct eyelid structure that Asians are born with. Deficiency of the attachment of the eyelid retractors, known as capsulopalpebral fascia, to the overlying skin and orbicularis oculi muscle allows the skin and muscle to roll upward, creating an overriding fold [2,7]. Other possible mechanisms may relate to hypertrophy of the orbicularis oculi muscle and coexisting epicanthus [8,9].

Generally, epiblepharon may resolve spontaneously with age as its prevalence has been reported to decrease from 46% in infants to about 2% in adolescents aged 13–18 [3,10]. It has been proposed that obesity may be attributable to the development of epiblepharon [2,11]. Hayasaka and colleagues reported that the body mass index (BMI) in Japanese 6- to 11-year-old children with epiblepharon was higher than that in children without epiblepharon [12]. By contrast, the association between obesity and epiblepharon was identified in Chinese boys aged 4 to 6 years [13]. Another previous study in Koreans found that only girls aged 12 to 15 years demonstrated significant correlation between the occurrence of symptomatic epiblepharon and body mass index [14]. The current literature provides evidence of a positive relationship between obesity and epiblepharon; however, these studies present inconsistencies in age and sex distributions. The discrepancies in previous findings regarding age distribution may originate from different population enrollments. The reference growth norm varies among ethnicities, and the relationship between epiblepharon and BMI in children of different ages remains to be clarified. Therefore, the present study sought to investigate the age- and sex-specific difference in the correlation between the incidence of epiblepharon and obesity in children and adolescents by using a population-based database.

## 2. Materials and Methods

### 2.1. Study Design and Data Source

This was a retrospective age-matched case–control study utilizing the Chang Gung Research Database (CGRD), the largest multi-institutional de-identified electronic medical records (EMR) database in Taiwan. Overall, the CGRD includes 21.2% of outpatients and 12.4% of inpatients in the Taiwanese population. The basic architecture of the CGRD contains most of the information from EMR for healthcare research. Owing to its high overall and disease-specific coverage, CGRD provides good access for clinical and scientific studies. Patient-level demographics and data on health conditions are coded according to the International Classification of Diseases, Ninth Revision, Clinical Modification (ICD-9-CM) codes before 2016 and ICD-10-CM codes after 2016 in the CGRD.

### 2.2. Patient Identification

**Figure 1:**
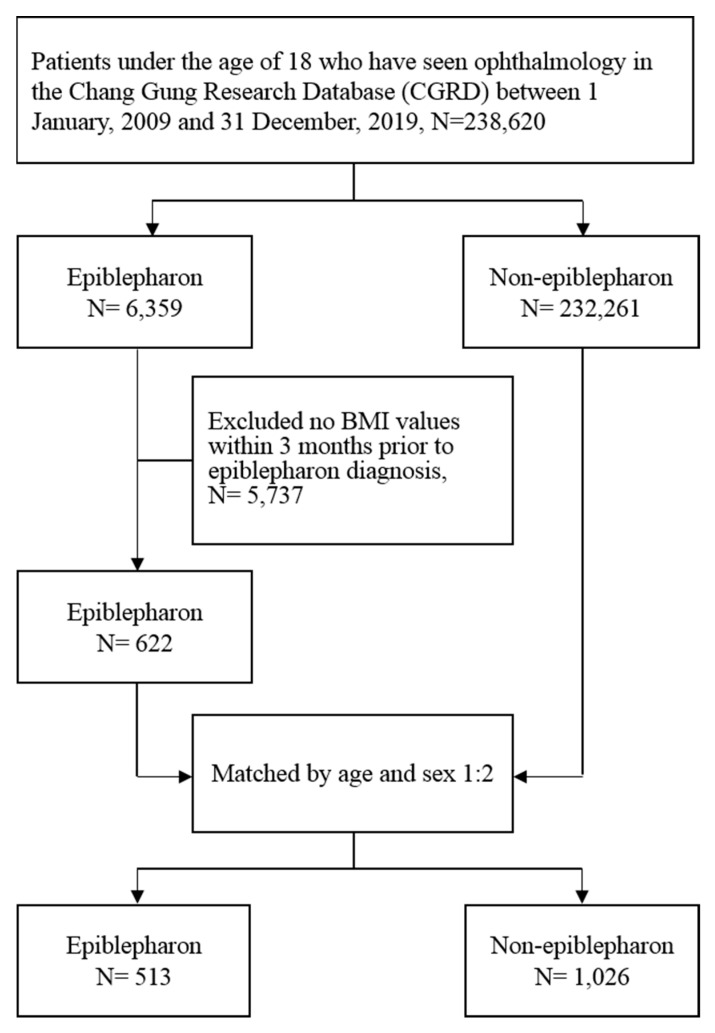
Flowchart of the study population selection.

We enrolled patients with epiblepharon aged ≤ 18 years from 1 January 2009 to 31 December 2019, by using epiblepharon-related diagnostic ICD-9-CM and ICD-10 codes: 374.00, 743.62, 743.63, H02.00, H02.05, Q10.2 and Q10.3. Patients with missing demographic information including body height and body weight, and those with specific congenital diseases including chromosomal abnormalities, congenital metabolic disorders, congenital ocular anomalies, congenital entropion, congenital ectropion, congenital malformations of the face and thyroid eye disease were excluded. For the comparison group, we randomly selected patients who did not receive a diagnosis of epiblepharon from CGRD, then matched them to the patients with epiblepharon by age and sex in a 2:1 ratio. Figure 1 demonstrates the flowchart of the study population selection. Furthermore, subjects were separated into 5 groups based on the following age ranges: 1–3 years, 4–6 years, 7–9 years, 10–12 years, and 13–18 years. We did not subdivide the 13–18 age group into two subgroups due to the smaller sample size, and the variation in puberty onset and changes in body composition among individuals.

### 2.3. Body Mass Index (BMI)

BMI was calculated as weight in kilograms divided by the square of height in meters. Body weight and height, obtained one month before and after the diagnosis of epiblpepharon, were used to calculate BMI and BMI percentile (adjusted for age and sex). Using standard age- and gender-specific BMI values specific to Taiwan norms, children and adolescents were categorized into three weight groups: normal weight (<85th percentile), overweight (85th to 95th percentile) and obese (≥95th percentile) [15].

### 2.4. Statistical Analysis

The baseline patient characteristics included age, sex, and BMI. In this study, categorical variables were reported as counts and percentages. Continuous variables were presented as median and interquartile range (IQR) if the distribution was skewed. Group comparisons were performed by Student’s *t*-test, chi-square test and Wilcoxon rank-sum test as appropriate. To compare the two groups in the epiblepharon and non-epiblepharon populations, the chi-square test was used for categorical variables and the independent samples *t*-test for continuous variables and baseline patient characteristics. To determine the association of obesity with outcomes of epiblepharon, the logistic regression analysis was adapted. A *p*-value < 0.05 was considered statistically significant. All the analyses were performed using SAS software version 9.4 (SAS Institute, Cary, NC, USA).

## 3. Results

According to the ICD diagnostic codes, from 1 January 2009 to 31 December 2019, a total of 513 children with epiblepharon and 1026 age-matched controls were included in this study. Of 513 children with epiblepharon, there were 296 boys (57.7%) and 219 girls (42.3%), while the control group comprised 592 boys and 434 girls. There was no significant difference in sex distribution in the two groups (*p* > 0.05). The mean age of these children was 6.28 ± 3.66 years. Table 1 shows the demographics and age distribution of the study population. Study subjects aged 1–3, 4–6, 7–9, 10–12 and 13–18 years were 24.6%, 37.2%, 19.3%, 10.7% and 8.2% of the population, respectively. Among children with epiblepharon, the distribution of BMI percentile classes was as follows: 27 (5.3%) were underweight, 280 (54.6%) were normal, 83 (16.2%) were overweight and 123 (24%) were obese. Compared with the control group, children with epiblepharon had a higher percentage of being overweight and obese. The median BMI value of the epiblepharon group (17.0 kg/m^2^, Q1–Q3 15.5–19.4 kg/m^2^) was significantly higher than that of the control group (16.3 kg/m^2^, Q1–Q3 15.0–18.1 kg/m^2^) (Table 1).

**Figure 2:**
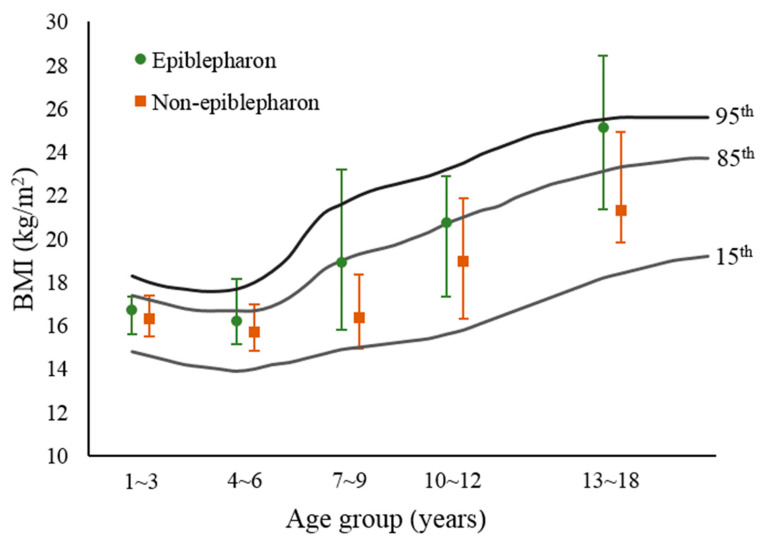
Comparison of body mass index (BMI) between epiblepharon and control groups (boys’ group). The background chart is the BMI-for-age growth curves by percentile, based on 2013 Taiwan standards.

**Figure 3:**
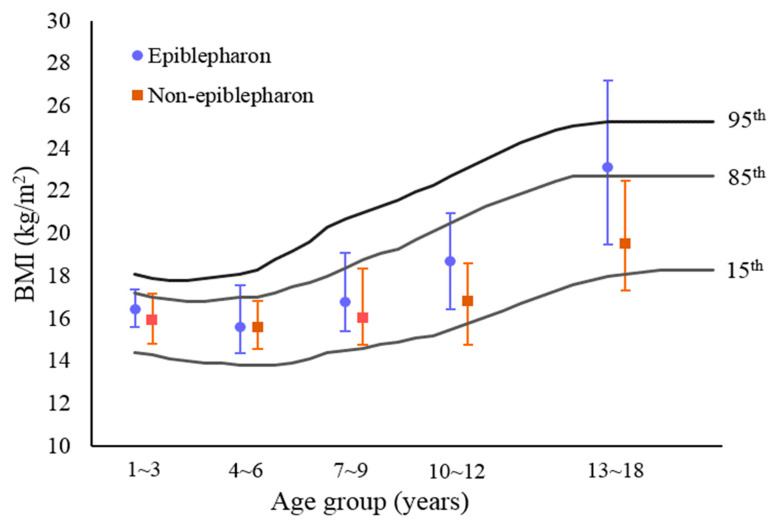
Comparison of body mass index (BMI) between epiblepharon and control groups (girls’ group). The background chart is the BMI-for-age growth curves by percentile, based on 2013 Taiwan standards.

Separate analyses were conducted for males and females, respectively, in different age groups. The distribution of children with and without epiblepharon according to age and sex is summarized in Table 2. Among boys with epiblepharon, a maximum of 107 patients (36.1%) were within the age range of 4 to 6 years, and the proportion decreased with age. For boys aged 4 to 9 years, the median BMI in children with epiblepharon was significantly higher than that in children without epiblepharon (*p* < 0.05). Additionally, the median BMI in boys aged 13 to 18 years was estimated to be statistically higher in the epiblepahron group compared with the control group (*p* = 0.045). Figure 2 demonstrated that boys with epiblepahron had BMI levels above the 85th percentile in three age groups (7–9, 10–12 and 13–18 years). On the other hand, 84 of 217 girls with epiblepharon (38.7%) were within the age range of 4 to 6 years, 45 (20.7%) at 7–9 years, 44 (20.3%) at 1–3 years, 18 (8.3%) at 10–12 years, and 26 patients (12%) at 13–18 years. An analysis showed no significant correlation between epiblepharon and high body mass index in three age groups (4 to 12 years), but 13- to 18-year-old girls with epiblepharon had significantly higher BMI values than those without epiblepharon (*p* < 0.05). The BMI level in 13- to 18-year-old girls with epiblepharon was above the 85th percentile (Figure 3).

The logistic regression analysis used for detecting relationships between epiblepharon and BMI was presented in Table 3. Overweight and obese boys who were 4 to 9 years of age had a higher risk of developing epiblepharon (OR: 1.74, 95% CI: 1.07–2.82, *p* = 0.025; OR: 3.06, 95% CI: 1.56–6.03, *p* = 0.001). As for overweight and obese girls, a higher risk of developing epiblepahron was noticed in those aged 4–6 years and 13–18 years (OR: 1.90, 95% CI: 1.07–3.38, *p* = 0.028; OR: 3.70, 95% CI: 1.38–9.97, *p* = 0.010).

## 4. Discussion

The results of our study indicate that overweight and obesity contribute to the occurrence of epiblepharon among the Taiwanese pediatric population. The median BMI measurements of children with epiblepharon is higher than those without epiblepharon in each age category. However, the effect of these associations varies with age and sex. To the best of our knowledge, this is the first study to elucidate the effect of age and its dependence on sex. 

Overweight and obesity not only increase the risk of many systemic diseases, but also adversely affect physical appearance, including the face and eyelids [16,17]. Recurrent lower eyelid entropion has been reported as a complication of morbid obesity [11]. The mass effect of the fatty and edematous tissue in the eyelid was recognized as the force that pushed the eyelid inward. Furthermore, several prior studies correlated obesity with epiblepharon. Hayasaka et al. stated that epiblepharon was significantly related to higher BMI in Japanese children aged 6–11 years [12]. For Japanese children with nephrotic syndrome receiving prolonged corticosteroid therapy, 46.7% of patients developing epiblepharon were overweight or obese [18]. Although the incidence of epiblepharon is relatively lower in Western countries, the association between obesity and epiblepharon has been noticed in a retrospective study conducted in the United States presenting that 40% of non-Asian children with epiblepharon had obesity, far exceeding the average rate of obesity among children [19].

Several studies have reported that the lower eyelid structure of Asians is histopathologically different from that of non-Asian people [20,21,22]. Microscopically, the capsulopalpebral fascia and the orbital septum at the inferior border of the tarsus do not have consistent fusion facilitating anterior and superior extension of the orbital fat in the lower eyelids [20,21,22]. With high-resolution magnetic resonance imaging (MRI), Carter et al. have shown that the orbital fat projecting forward and upward in all Asian lower eyelids rolls up the anterior orbicularis oculi muscle, giving the eyelid a bulging appearance [23]. For obese children, excess submuscular fat pad in the zygomatic region and lower eyelid pushes the eyelid margin inward, aggravating epiblepharon [22,23]. However, the eyelid fascia structure does not change with growth [22]. If children remain obese in adolescence, the excess subcutaneous fat probably continues to affect the structure of the eyelid. Similarly, our study demonstrated that the risk of epiblepahron increased 2–4 times for overweight and obese adolescents.

In the present study, a higher incidence of epiblepharon in overweight and obese boys was noticed, even though prior studies presented no sexual predilection with respect to the incidence of epiblepharon [3,10,12]. In Taiwan, the overall prevalence of obesity (including overweight) was higher in boys than in girls [24]. In the 2000s, the obesity rate was 14.6% among elementary school children (boys 17.0%, girls 12.0%) and 16.9% (boys 20.0%, girls 13.4%) among middle school students [25]. The result of our study was in line with the study conducted by Oshima et al. who reported that epiblepharon was more prevalent in Japanese boys than in girls [26]. A cross-sectional study conducted by Zhou et al. also showed that young boys had a higher risk of developing epiblepharon than young girls among Chinese preschoolers aged 3–6 years [27].

Different moderation effects of obesity on epiblepharon were observed in the current study. A higher risk of developing epiblepharon among overweight and obese children was encountered in boys aged 4–9 years (OR = 1.74, 95% CI = 1.07–2.82, *p* = 0.025; OR = 3.06, 95% CI = 1.56–6.03, *p* = 0.001) and in girls aged 13–18 years (OR = 3.70, 95% CI = 1.38–9.97, *p* = 0.01). The effect of obesity contributing to the development of epiblepharon appeared earlier in boys and later in girls. This age disparity between male and female populations in our study coincides with previous studies. Yan et al. investigated children below the primary school age and found a significantly higher BMI among boys aged 4–6 years [13]. Moreover, Ahn et al. indicated that 12- to 15-year-old girls with epiblepharon had significantly higher BMI levels [14].

Epiblepharon often becomes less pronounced with age as a result of the growth of the facial and orbital skeleton [28]. A decreasing trend exists in our study as the percentage of epiblepharon declines with age from 37.2% to 8.2%. Likewise, a previous Japanese study estimated that only 28 of 1402 teens aged 16 to 18 years (1.9%) had epiblepharon [3]. Nevertheless, the association between epiblepharon and obesity became more prominent in the female population at the age ≥ 13 years in the present study. Previous studies also found that congenital epiblepharon was more persistent in females [14,29]. Ahn et al. reported that obese Korean girls at 12 to 15 years of age demonstrated symptomatic epiblepharon at a significant level [14]. The sex difference in skeleton growth and hormonal changes is probably a reflection of the revelation [14]. In addition to the anatomical factors, some researchers addressed that the influence of hormones differs by sex during puberty [14]. Females tend to accrue more adipose tissue in the subcutaneous depot in response to sex steroids, which may aggravate the development of epiblepharon [30,31,32]. Nonetheless, this hypothesis has not been completely elucidated yet.

On the basis of our findings, indications for the management of epiblepharon in overweight and obese children should be different for boys and girls. Likewise, Valencia et al. found that the distribution of Japanese patients who required surgical correction for epiblepharon was associated with age and sex [29]. The majority of patients sought eyelid correction surgery at 4–12 years of age. Among patients aged ≥ 13 years who underwent correction surgery, the percentage was significantly higher in females than that in males. Consequently, given the relationship between obesity and epiblepharon, ophthalmologists and pediatricians can pay more attention to the appearance of the eyelid when encountering children with high BMI.

Our study has several strengths and limitations. This database research contains a large number of cases from multiple hospitals in Taiwan. To our knowledge, this is the first study to investigate the association between obesity and epiblepharon in children with the broadest range of age covering 1 to 18 years, allowing us to clarify the impact of obesity on epiblepharon in different age groups. However, the retrospective nature restricted a detailed review on severity grading, ocular symptoms and refractive data, which are unavailable in the claims database. Moreover, epiblepharon may have been underdiagnosed in this population because many children with epiblepharon are asymptomatic, causing potential bias. In fact, there is no clear diagnostic code for epiblepharon. Our results were analyzed according to the ICD codes recommended by the American Academy of Ophthalmology, which was considered the most appropriate coding [33]. Although we used big data for research, the sample size of adolescents was not enough, causing the difference in BMI levels to be not statistically significant between boys with epiblepharon aged ≥ 13 years and those without epiblepharon. Therefore, further studies are required to clarify the sex-based differences in the underlying mechanisms responsible for the development of epiblepharon in the overweight and obese pediatric population.

## 5. Conclusions

In summary, our study demonstrates a significant correlation between the incidence of epiblepahron and obesity in Taiwanese children, suggesting that higher BMI may be an important aggravating factor of epiblepharon. The moderation effect of obesity on epiblepharon appears earlier in boys and later in girls. Sexual dimorphism in the age distribution of the association between obesity and epiblepharon implies distinct pathophysiologies of epiblepharon in both sexes. Given the rise in the prevalence of childhood obesity, it is prudent to be aware of its association with epiblepharon. The findings of the present study provide clinicians with cogent evidence for a more personalized approach to epiblepharon.

## Figures and Tables

**Table 1:** Demographic characteristics of patients.

Characteristic	Epiblepharon	Control	*p*-Value
Sex, *n* (%)			1.000
Female	217 (42.3)	434 (42.3)	
Male	296 (57.7)	592 (57.7)	
Age, years			
Mean ± SD (range)	6.28 ± 3.66 (1–18)	6.28 ± 3.66 (1–18)	1.000
Age at baseline, years			1.000
1–3	126 (24.6%)	252 (24.6%)	
4–6	191 (37.2%)	382 (37.2%)	
7–9	99 (19.3%)	198 (19.3%)	
10–12	55 (10.7%)	110 (10.7%)	
13–18	42 (8.2%)	84 (8.2%)	
BMI classification			<0.001
Normal	307 (59.8%)	746 (72.7%)	
Overweight	83 (16.2%)	126 (12.3%)	
Obese	123 (24.0%)	154 (15.0%)	
BMI, median (Q1–Q3)	17.0 (15.5–19.4)	16.3 (15.0–18.1)	<0.001

**Table 2:** The association between epiblepharon and body mass index (BMI) by sex and age.

Age (y)	Epiblepharon	Male			Female		
		No.	BMI, Median (Q1–Q3)	*p*-Value	No.	BMI, Median (Q1–Q3)	*p*-Value
1–3	Yes	82	16.7 (15.6–17.3)	0.195	44	16.5 (15.6–17.4)	0.034
	No	164	16.3 (15.5–17.4)		88	15.9 (14.8–17.2)	
4–6	Yes	107	16.2 (15.1–18.1)	0.012	84	15.6 (14.4–17.6)	0.366
	No	214	15.7 (14.8–17.0)		168	15.6 (14.6–16.8)	
7–9	Yes	54	18.9 (15.8–23.2)	0.001	45	16.8 (15.4–19.1)	0.169
	No	108	16.4 (14.9–18.4)		90	16.0 (14.8–18.4)	
10–12	Yes	37	20.7 (17.3–22.9)	0.289	18	18.7 (16.4–21.0)	0.060
	No	74	19.0 (16.3–21.9)		36	16.8 (14.8–18.6)	
13–18	Yes	16	25.1 (21.4–28.4)	0.045	26	23.2 (19.5–27.2)	0.008
	No	32	21.3 (19.8–24.9)		52	19.5 (17.3–22.5)	

**Table 3:** Odds ratios (OR) between epiblepharon and overweight/obesity by sex and age.

Age (y)	Overweight/ Obesity	Male					Female				
		Epiblepharon	Control				Epiblepharon	Control			
		N (%)	N (%)	OR	95% CI	*p*-Value	N (%)	N (%)	OR	95% CI	*p*-Value
1–3	Yes	21 (25.6)	38 (23.2)	1.14	0.62–2.11	0.673	15 (34.1)	21 (23.9)	1.65	0.75–3.65	0.216
	No	61 (74.4)	126 (76.8)				29 (65.9)	67 (76.1)			
4–6	Yes	45 (42.1)	63 (29.4)	1.74	1.07–2.82	0.025	30 (35.7)	38 (22.6)	1.90	1.07–3.38	0.028
	No	62 (57.9)	151 (70.6)				54 (64.3)	130 (77.4)			
7–9	Yes	31 (57.4)	33 (30.6)	3.06	1.56–6.03	0.001	14 (31.1)	24 (26.7)	1.24	0.57–2.72	0.589
	No	23 (42.6)	75 (69.4)				31 (68.9)	66 (73.3)			
10–12	Yes	18 (48.7)	29 (39.2)	1.47	0.66–3.26	0.343	7 (38.9)	6 (16.7)	3.18	0.88–11.56	0.079
	No	19 (51.4)	45 (60.8)				11 (61.1)	30 (83.3)			
13–18	Yes	10 (62.5)	14 (43.8)	2.14	0.63–7.33	0.225	15 (57.7)	14 (26.9)	3.70	1.38–9.97	0.010
	No	6 (37.5)	18 (56.3)				11 (42.3)	38 (73.1)			

## Data Availability

The data presented in this study are available upon request from the corresponding author. The data are not publicly available due to ethical restrictions.
